# Multiple latent variables but functionally dependent output mappings underlying the recognition of own- and other-race faces for Chinese individuals: Evidence from state-trace analysis

**DOI:** 10.3389/fpsyg.2022.968956

**Published:** 2022-07-28

**Authors:** Wei Liu, Yuxue Jia

**Affiliations:** ^1^Department of Educational Science, Anshun University, Anshun, China; ^2^Faculty of Human Ecology, Universiti Putra Malaysia, Serdang, Selangor, Malaysia

**Keywords:** face inversion effect, face recognition, state-trace analysis, other-race effect, latent variable, functional dependence

## Abstract

To explore the number of latent variables underlying recognition of own- and other-race faces for Chinese observers, we conducted a study-recognition task where orientation, stimuli type, and duration were manipulated in the study phase and applied state trace analysis as a statistic method. Results showed that each state trace plot on each pair of stimuli types matched a single monotonic curve when stimuli type was set to state factor, but separate curves between face and non-face showed up when the state factor was orientation. The results implied that at least two latent variables affected recognition performance in the inversion paradigm. Besides, the unidimensional structure between own- and other-race faces regardless of the state factor suggested that Chinese participants used the same recognition mechanism for unfamiliar own- and other-race faces in the inversion paradigm.

## Introduction

One of the most robust phenomena in face processing is the face inversion effect (FIE; Yin, 1969), which indicates that inversion reduces recognition performance on faces more than on non-face objects (Rossion, 2008; McKone and Yovel, 2009). It was argued that inversion harmed the configural information which was important for faces other than non-face objects, resulting in a qualitative shift in face processing strategy (Rossion, 2008). Although there was a clear distinction between the inversion effect for unfamiliar non-face stimuli and faces, some studies suggested that the inversion effect for non-face objects could increase with expertise to the same degree as the inversion effect for faces (Diamond and Carey, 1986; Gauthier and Tarr, 1997; Campbell and Tanaka, 2018). Because expertise is primarily acquired through experience, it was wondered if contact experience would affect the inversion effect between different faces, such as those of one’s race and those of another race.

Most people have more experience contacting with own- than other-race individuals, especially those who have lived in their homeland all along since their birth. Numerous research has shown that people recognize upright faces of their race better than faces of other races, a phenomenon known as the other-race effect (ORE; Meissner and Brigham, 2001). As for inverted faces, several research found a larger FIE for own- than other-race faces, implying the benefit of configural processing for own-race faces (Rhodes et al., 1989; Sangrigoli and de Schonen, 2004; Hancock and Rhodes, 2008; Megreya et al., 2011). Other studies, on the other hand, found that there was no difference in inversion decrement between own- and other-race faces (Buckhout and Regan, 1988; Rhodes et al., 2006; Matheson et al., 2012), or even that the inversion effect was larger for other-race faces than for own-race faces (Valentine and Bruce, 1986).

Because of the mixed results of the inversion effect on other-race faces, it was difficult to determine if participants used the same amount of configural and featural information to process own- and other-race faces. Furthermore, because the traditional analysis of variance (ANOVA) used in the aforementioned studies was not a direct measurement of the underlying structure, the findings were insufficient to conclude that there were two latent variables (configural and featural information) affecting the own- and other-race faces differently.

A statistic method called state trace analysis (STA) has been developed to directly analyze the underlying structure of a phenomenon (Bamber, 1979; Prince et al., 2012; Dunn and Kalish, 2018). This method suggests that the dependent variables are directly regulated by unobserved latent variables which are affected by manipulated independent variables. The mappings of latent variables to dependent variables are called output functions, and the mappings of independent variables to latent variables are called input functions. Specifically, if there is only one latent variable influencing two dependent variables and the output functions are monotonic, the plot of the two dependent variables against each other should be a single monotonic curve because we can invert one of the monotonic output function and nest it into the other. On the other hand, if non-monotonic curves or separate curves are observed, it can be reasonably assumed that there are multiple latent variables. In short, a scatter plot between two dependent variables (or a dependent variable in two conditions), known as a state trace plot, is the core of STA. The number of latent variables can be inferred from the monotonicity of the curve fitted by the dots on the plot. Particularly, if the curve is not monotonic, then a model with a single latent variable will be rejected, implying that there are at least two latent variables affecting the dependent variables. However, it should be noted that a single monotonic curve on a state trace plot cannot prove the existence of a single latent variable structure, as this type of plot can also be produced by multiple latent variables with functional dependence between each pair of input functions or between output functions (Dunn et al., 2014; Dunn and Kalish, 2018).

In terms of detecting the number of latent variables, STA was argued to be more advantageous than traditional analysis of variance (Newell and Dunn, 2008; Dunn and Kalish, 2018). First, ANOVA is strictly based on the linearity assumption which is hard to confirm, while STA only assumes that the output function is monotonic, which is laxer than linearity (Dunn and Kalish, 2018). Additionally, significant ANOVA interaction effects are usually considered an indicator of functional dissociation, the classical logic for determining distinct underlying processes. Numerous researchers, however, have disputed this logic, claiming that neither single nor double dissociation can reveal multiple underlying processes (Loftus, 1978; Dunn and Kirsner, 1988, 2003; Dunn, 2003; Davies, 2010; Wagenmakers et al., 2012).

However, whether STA can be used to determine the number of underlying processes or systems is still under dispute. Some researchers argue that this question is beyond the scope of STA (Ashby, 2014, 2019; Ashby and Bamber, 2022), while others believe that the number of latent variables is the lower bound of the number of processes or systems (Dunn et al., 2014; Dunn and Kalish, 2018; Stephens et al., 2020). This debate will continue until the term “process” or “system” in psychology is clearly defined (Dunn et al., 2014; Dunn and Kalish, 2018). Regardless of their differences, they both concur that STA can be used to determine the number of latent variables or parameters. As a result, we will refer to a latent variable instead of a process or system in this article.

In the field of face recognition, several studies applied this method to explore the latent variables underlying FIE and found that the state trace plot was a monotonic curve between non-face objects (house) and unfamiliar faces (Loftus et al., 2004; Prince and Heathcote, 2009), suggesting that there were no differences in configural and featural processing between the two types of stimuli. However, in these STA studies, the quantitative statistical method for STA was Spearman rank correlation, which was insufficient for inferring monotonicity due to the lack of consideration of sampling error (Kalish et al., 2016). Several techniques for quantitative STA have recently been developed (Prince et al., 2012; Kalish et al., 2016; Benjamin et al., 2019), one of which is based on conjoint monotonic regression (CMR; Kalish et al., 2016). CMR compares the goodness-of-fit between a partial order model, in which the x-coordinates have the same partial order as the y-coordinates for all dots, and a monotonic model, in which the two coordinates have the same order as well as monotonically related to each other. Because the monotonic model is nested within the partial order model, if the monotonic model is true, the difference in fits between the two models is small. As a result, if we calculate a large number of differences in fits using bootstrap samples and see how many of them are larger than the original one based on raw data, the proportion will be high. The null hypothesis that the monotonic model is true can be rejected if the proportion is less than the criterion, for example, 0.05. The CMR Software Package is available for download on github^1^ in both R and MATLAB versions (Dunn and Kalish, 2018).

In this study, we aimed to replicate the previous studies (Loftus et al., 2004; Prince and Heathcote, 2009) by using CMR and to further investigate the difference between using configural and featural information of own- and other-race faces.

## Materials and methods

### Participants

Using G*Power, we found that at least 43 participants were required for detecting the interaction between orientation and stimuli type with a medium effect size *f* = 0.25 and 95% statistical power in this repeated measure design. Since ANOVA was not the main statistical method of this experiment, we decided to use a slightly larger sample size. Therefore, 49 Chinese undergraduate students with normal or corrected-to-normal vision at Anshun University were included in this study (10 males, aged from 19 to 23 with a mean of 20.51). Participants were native Chinese and reported no experience contacting other-race individuals, as well as living or traveling abroad. All the participants were enrolled through an online recruitment program reviewed and approved by the student affairs department of Anshun University. The study had been reviewed and approved by the College Research Ethics Committee of Anshun University. Each participant had signed informed consent before the experiment and received course credit as compensation.

### Stimuli

**FIGURE 1:**
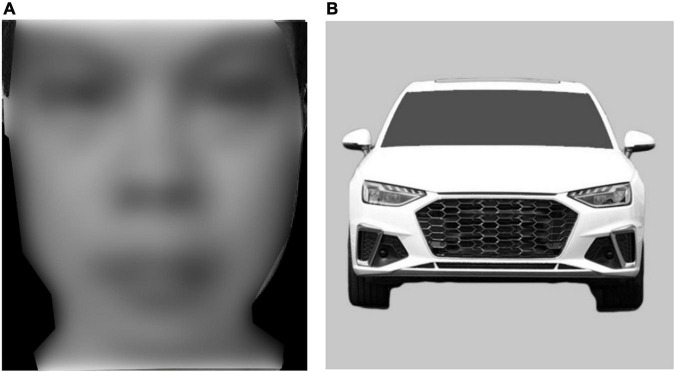
Examples of stimuli. **(A)** Face. **(B)** Car front pictures. The example of the face is one of the authors who has authorized the usage of the face. This face is not used in the experiment, but to show the sample of our stimuli.

The 100 Asian faces were from the Taiwan Facial Expression Image Database (TFEID; Chen and Yen, 2007) and 100 Caucasian faces were from the Chicago face database (CFD; Ma et al., 2015). All the faces were males with neutral expressions. Pictures were then cropped to 260 × 300 pixels without hair, ears, and clothes, and converted to black and white by photoshop cs6 (Figure 1A). Car front pictures were used as non-face stimuli like in previous studies (Tanaka and Gauthier, 1997; Wallace et al., 2008) because their configural homogeneity and exposure to individuals were very close to faces. About 100 different white car front pictures were selected from the Chinese automobile websites.^2^,^3^ All the cars were common to be seen in the street. Car front images were cut out from the raw pictures, resized to 500 × 500 pixels, and then converted to black and white. To eliminate the cues of context, logo, and license plate, we set the color of the background to RGB (200, 200, 200) and the front windshield to RGB (80, 80, 80), and erased the logo and license plate (Figure 1B).

### Design and procedure

We conducted a 3 (stimuli type: Asian face, Caucasian face, Car front) × 2 (orientation: upright, inverted) × 5 (duration: 0.1, 0.25, 1, 3, 5 s) repeated measure design. All three independent variables were within-subject factors. It is noticeable that we used a longer duration than in the previous face-inversion STA studies (Loftus et al., 2004; Prince and Heathcote, 2009, 2010) because we wanted to extend the result to commonly used exposure time (3–5 s) in inversion paradigm (e.g., Yin, 1969; Scapinello and Yarmey, 1970; Diamond and Carey, 1986; Valentine and Bruce, 1986; Cabeza and Kato, 2000; Freire et al., 2000; Civile et al., 2016; Klargaard et al., 2018).

About 100 images in each stimuli type were equally divided into five groups at random for every participant. Therefore, different participants used different groupings. Every participant should complete 15 blocks of study-recognition tasks. The first block was always Asian face, followed by a car front block and then a Caucasian face block. The same order was repeated five times. There was a 30-s blank screen interval between blocks. In each block, 10 images were randomly selected and presented one after another as targets in the study phase, corresponding to the 10 combinations of orientation and duration. After the study phase, there was a 30-s blank screen, followed by the test phase with all the 20 upright pictures in that group, presented sequentially and randomly. Participants were asked to rate their confidence in each of the shown pictures from 1 (definitely new) to 4 (definitely old). As a result, each participant should memorize 150 images (10 trails for each block) and make 300 confidence ratings (20 trails for each block) in total.

Each study trial began with a 1,000-ms fixation cross in the center of the screen. Next came the target picture in its assigned orientation for its assigned duration, and then the blank screen for 1,000 ms. The pictures in the test phase were always presented on the screen until response. Rating scores and reaction time were recorded. The whole experiment needed approximately 60 min on average.

This experiment was programmed by Psychopy3 (Peirce et al., 2019) and was run on laptops with a resolution of 2,160 × 1,440 pixels. The size of the images was set to approximately 6.5° (horizontal) × 7.5° (vertical) for faces and 7.5° × 7.5° for cars at a viewing distance of about 50 cm.

## Results

We used the same procedure of Loftus et al. (2004) to transform the raw rating data to recognition performance (*p*_*r*_). First, the rating response of 1–4 was assigned to values of 0–3, respectively. Next, 11 mean confidence ratings for each stimuli type were computed from the transformed value: 10 for targets in the 10 study conditions and one for the distractors. And then the mean confidence ratings were divided by three yielding ratios for targets and distractors that were similar to hit rates (*p*_*hit*_) and false-alarm rates (*p*_*fa*_), respectively. Finally, the *p*_*r*_ for each condition could be calculated using the formula below:


$$p_{r}=\frac{p_{h\,i\,t}-p_{f\,a}}{1-p_{f\,a}}$$

### Analysis of variance in recognition performance

A 3 (stimuli type) × 2 (orientation) × 5 (duration) repeated measure analysis of variance (ANOVA) in *p*_*r*_ revealed significant main effect of orientation, *F(1, 48)* = *185.98*, *p* < *0.001*, η*_*p*_*^2^ = *0.80*; participants recognized upright images better than inverted images. A significant main effect of duration was also found, *F(4, 192)* = *27.03*, *p* < *0.001*, η*_*p*_*^2^ = *0.36.* The interaction between orientation and stimuli type was significant, *F(2, 96)* = *15.28*, *p* < *0.001*, η*_*p*_*^2^ = *0.24*, as well as the interaction between orientation and duration, *F(4, 192)* = *8.45*, *p* < *0.001*, η*_*p*_*^2^ = *0.15*. The main effect of stimuli type, *F(2, 96)* = *2.03*, *p* = *0.137*, the interaction between stimuli type and duration, *F(8, 384)* = *0.73*, *p* = *0.666*, as well as the three-way interaction among stimuli type, orientation, and duration, *F(8, 384)* = *1.54*, *p* = *0.142*, failed to reach significance.

**FIGURE 2:**
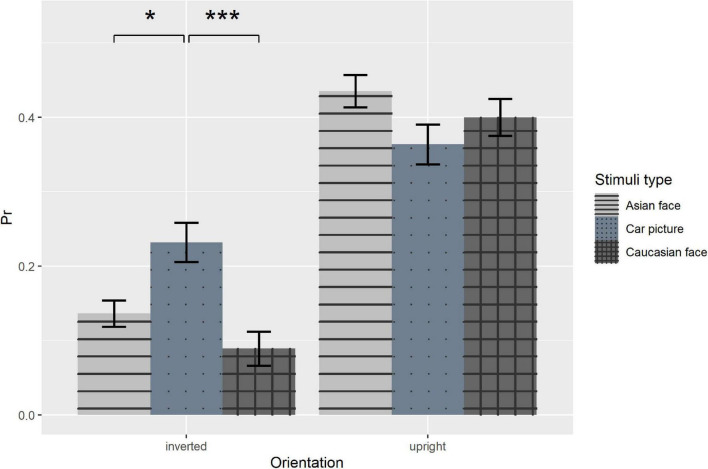
Recognition performance as a function of orientation and stimuli type. Error bars represent standard errors. *p < 0.05, ***p < 0.001.

To further explore the interaction between orientation and stimuli type, we compared each pair of stimuli types in each condition. In upright condition, the pairwise comparisons with Bonferroni adjustment showed that there were no differences of recognition performance between Asian faces and Cars, *t(48)* = *2.19, p* = *0.100*, between Caucasian faces and Cars, *t(48)* = *0.90, p* > *0.999*, as well as between Asian faces and Caucasian faces, *t(48)* = *1.51, p* > *0.416*, indicating that we did not find a significant other-race effect, though the *p*_*r*_ was slightly higher for Asian faces (mean = 0.44, 95% CI = [0.38, 0.49]) than Caucasian faces (mean = 0.40, 95% CI = [0.33, 0.47]). On the other hand, in inverted condition pairwise comparisons with Bonferroni adjustment revealed that the recognizing performance of car pictures was significantly better than own-race faces, *t(48)* = *2.74, p* = *0.026, Cohen’s d* = *0.21*, and other-race faces, *t(48)* = *3.98, p* < *0.001, Cohen’s d* = *0.31* (Figure 2). The results implied that inversion produced disproportionately larger decrease in recognition performance for faces than cars, namely, the face inversion effect. To further test this, we subtracted *p*_*r*_ in inverted conditions from upright conditions in each combination of stimuli type and duration and used this value as the index of inversion effect in a 3 (stimuli type) × 5 (duration) repeated measure ANOVA. The main effect of stimuli type was significant, *F(2, 96)* = *15.28*, *p* < *0.001*, η*_*p*_*^2^ = *0.24*. Pairwise comparison with Bonferroni adjustment showed that the inversion effect of car pictures was significantly smaller than own-race faces, *t(48)* = *4.46, p* < *0.001, Cohen’s d* = *0.29*, as well as other-race faces, *t(48)* = *4.36, p* < *0.001, Cohen’s d* = *0.31*, but there was no difference in inversion effect between faces. These findings supported the existence of the classical FIE. The main effect of duration was also significant, *F(4, 192)* = *8.45*, *p* < *0.001*, η*_*p*_*^2^ = *0.15*. Pairwise comparison with Bonferroni adjustment showed that the inversion effect at 0.1 s duration was significantly smaller than those at 1 s duration, *t(48)* = *4.03, p* = *0.002, Cohen’s d* = *0.34*, at 3 s duration, *t(48)* = *4.39, p* < *0.001, Cohen’s d* = *0.36*, and at 5 s duration, *t(48)* = *4.48, p* < *0.001, Cohen’s d* = *0.44.* The interaction between stimuli type and duration was not significant, *F(8, 384)* = *1.54*, *p* = *0.142*.

**FIGURE 3:**
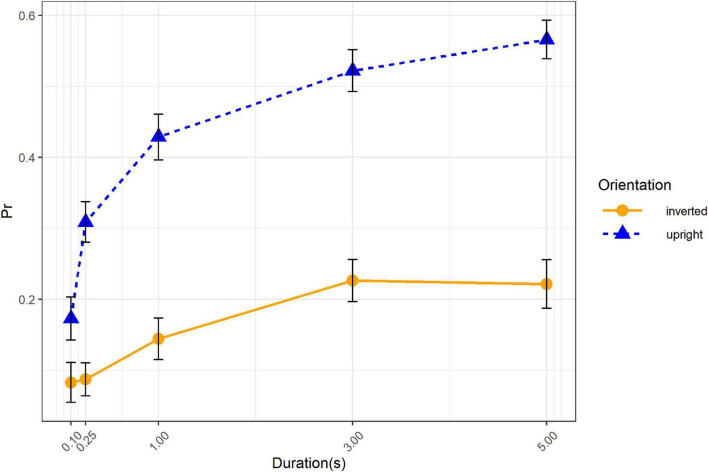
Recognition performance as a function of orientation and duration. Error bars represent standard errors.

The simple effects tests of orientation × duration interaction showed that the effect of duration was significant in upright condition, *F(4, 48)* = *26.22*, *p* < *0.001*, η*_*p*_*^2^ = *0.69*, as well as in inverted condition, *F(4, 48)* = *4.64*, *p* = *0.003*, η*_*p*_*^2^ = *0.28* (Figure 3). Polynomial contrast of duration revealed that the linear trend was significant in both upright, *t(48)* = *10.18, p* < *0.001*, and inverted condition, *t(48)* = *3.59, p* < *0.001*. Recognition performance in both orientations increased with the duration getting longer. Given that the recognition performance increased from 0.17, 95% CI = [0.10, 0.24], to 0.57, 95% CI = [0.49, 0.64], for upright condition and from 0.08, 95% CI = [0.02, 0.14], to 0.22, 95% CI = [0.14, 0.30], for inverted condition, we then assumed that the interaction might be due to the influence of duration on upright condition was larger than in inverted condition.

### Analysis of variance in reaction time

A 3 (stimuli type) × 2 (orientation) × 5 (duration) repeated measures analysis of variance (ANOVA) in reaction time showed that the main effect of orientation was significant, *F(1, 48)* = *5.63*, *p* = *0.022*, η*_*p*_*^2^ = *0.11*. Response to inverted pictures was slower than upright pictures. The main effect of stimuli type was also significant, *F(2, 96)* = *7.49*, *p* < *0.001*, η*_*p*_*^2^ = *0.14*. Pairwise comparison with bonferroni adjustment revealed that participants were slower with Asian faces than car pictures, *t(48)* = *3.47, p* = *0.003, Cohen’s d* = *0.18*, and Caucasian faces, *t(48)* = *3.98, p* < *0.001, Cohen’s d* = *0.21*. These results suggested that the classical FIE observed in *p*_*r*_ was not due to speed accuracy trade-offs.

### State-trace analysis in recognition performance

We found the classical FIE from ANOVA; however, the result was strictly based on linearity assumption which was hard to confirm. Besides, the logic of dissociation indexed by significant interaction was argued to be problematic (Dunn and Kirsner, 1988, 2003; Davies, 2010). Thus, the classical FIE was insufficient to conclude that recognizing faces and non-faces was disproportionately influenced by multiple latent variables. Some researchers then embraced STA for evaluating the number of latent variables (e.g., Newell and Dunn, 2008; Dunn et al., 2012; Hamm, 2014; Stephens et al., 2019).

Therefore, to directly detect the number of latent variables underlying recognition performance, we conducted three quantitative state-trace analyses by CMR on each pair of stimuli types in *p*_*r*_. In each of the three STA, the recognition performance on stimuli type was used as the state factor such as in previous research (Loftus et al., 2004; Prince and Heathcote, 2009), which constituted the two axes of each state trace plot. The points on the scatter plot were formed by the trace and dimension factors, which were duration and orientation, respectively. We set the partial order constraints in CMR based on the results of previous ANOVA, with upright pictures performing better than inverted ones and longer duration associated with higher performance.

**FIGURE 4:**
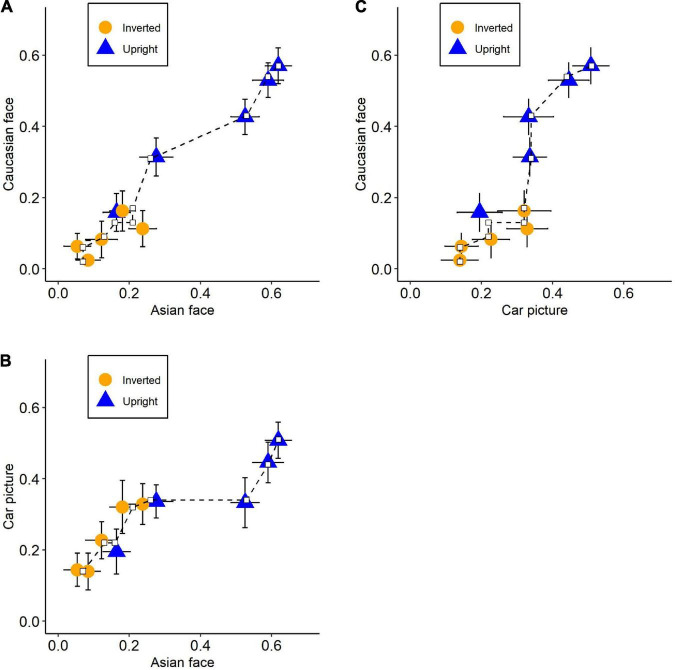
State trace plots for each pair of stimuli types. The axis represents the recognition performance (*p*_*r*_) of its corresponding stimuli types. Plots between recognition performance on **(A)** Asian faces and Caucasian face, **(B)** Asian faces and Car front pictures, and **(C)** Caucasian faces and Car front pictures. Orange circles: inverted condition; Blue triangles: upright condition. Error bars indicate standard errors and dash lines represent the best-fitting monotonic curve.

Each of the three-state trace plots demonstrated that the points could be fitted by single monotonically increasing curves (Figure 4). Null hypothesis significance testing of CMR based on 10,000 bootstrap samples revealed that the fit-value (effect size of CMR; Kalish et al., 2016) and *p*-value were *fit* = *0.17* and *p* = *0.671* for Asian-Caucasian plot, *fit* = *0.64* and *p* = *0.481* for Caucasian-Car plot, and *fit* = *0.47* and *p* = *0.363* for Asian-Car plot. Consistent with previous studies (Loftus et al., 2004; Prince and Heathcote, 2009), these results indicated that the uni-dimensional structure could not be rejected, implying only one latent variable regulating the performance among the three stimuli types. The functional dependence of input mappings or output mappings, however, might theoretically also account for this one-dimension state trace (Dunn and Kalish, 2018). Loftus et al. (2004) embraced functional dependence to explain their results. They assumed that recognition performance was influenced by two latent variables: featural strength and configural strength. The former was unaffected by stimuli type, while the latter had an equal impact on non-face objects (house) and unfamiliar faces. Therefore, the output mapping from latent variables to recognition performance for the two stimuli types was functionally dependent, resulting in a single monotonic curve on the state trace plot.

Loftus et al. (2004) gave up the possibility of one latent variable because numerous studies suggested that there were two kinds of information affecting face recognition (Rossion, 2008; McKone and Yovel, 2009). We noticed that this possibility could also be ruled out by an additional STA in which orientation was used as a state factor. According to their model, if orientation was used as a state factor, the state trace plot between upright and inverted conditions should be separate curves. Since Loftus et al. (2004) did not conduct the STA between upright and inverted conditions, we thought it was necessary to implement it for further exploration.

**FIGURE 5:**
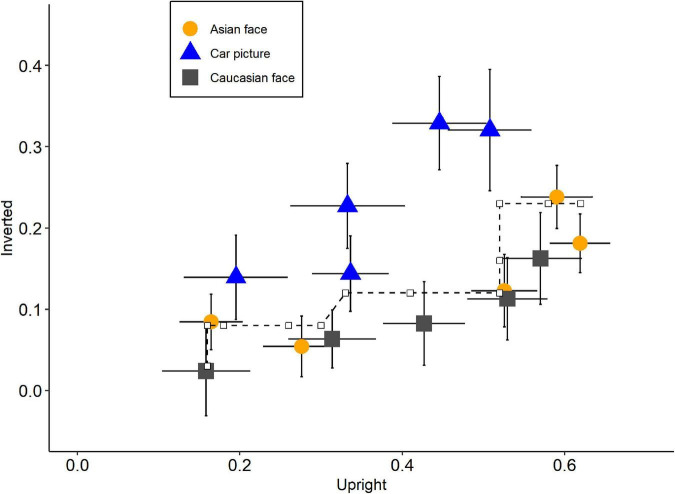
State trace plot with orientation as state factor. The axis represents the recognition performance (*p*_*r*_) of its corresponding stimuli types. Orange circles: Asian faces; Blue triangles: Car front pictures; Gray squares: Caucasian faces. Error bars indicate standard errors and dash line represents the best-fitting monotonic curve.

We performed another STA with orientation as a state factor. The partial order constraints, in this case, were longer duration associated with higher performance. Results showed that the uni-dimensional structure was rejected in this situation, *p* = *0.017, fit* = *14.12* (Figure 5). Notably, the traces of Asian and Caucasian faces were extremely close to each other, and both were apart from car pictures. As a result, the violation of uni-dimensional structure was most likely due to the different trends between faces and cars. To further test this, we split the analysis into three sub-analyses, each with only two stimuli types as the levels of dimension factor. The results were as expected. The significant multi-dimensional structure was detected between Asian faces and car pictures, *p* = *0.001, fit* = *9.91*, as well as between Caucasian faces and car pictures, *p* = *0.010, fit* = *8.80*, while the Asian-Caucasian plot suggested a uni-dimensional structure, *p* = *0.952, fit* = *0.016*. These results supported the two-latent variable claim. Interestingly, the state trace plot between Asian and Caucasian faces remained a single monotonic curve and this would be discussed in the next section.

## Discussion

We believed that running two STAs on the same data with different state factors would be a good way to test the number of latent variables and check the functional dependence between input functions. First, it was logically assumed that if there was only one latent variable between the independent and dependent variables, one of the monotonic output functions could always be inverted and then nested into the other, resulting in a single monotonic curve on state trace plot regardless of the state factors used. Besides, if multiple latent variables were functionally dependent for each pair of input mappings, all of the latent variables could be regarded as being controlled by a single virtual independent variable (Dunn et al., 2014), which would also produce a single monotonic curve on the state trace plot regardless of the state factor used. Furthermore, if the single monotonic curve was due to functional dependence between output mappings with multiple latent variables, using another state factor would probably turn the plot into non-monotonic curves. In other words, a single-latent-variable model and functionally dependent input functions could both be ruled out, and the single monotonic curve could be attributed to functionally dependent output mappings if non-monotonic curves were observed in another STA with a different state factor using the same data.

For example, in the model proposed by Loftus et al. (2004), the output function was as follows:


$$p_{i\,j\,k}=\left[F_{i\,j}+\left(1-F_{i\,j}\right)\,C_{j\,k}\right]\,Y_{k},$$

where *p* was recognition performance; *F* and *C* were two latent variables, featural strength and configural strength, respectively; *Y* was free parameters differed for stimuli types; and the subscripts *i*, *j*, and *k* denoted different levels of duration, orientation (*i* = *U*, upright; *i* = *I*, inverted), and stimuli type (*k* = *F*, Face; *k* = *N*, Non-face), respectively. Besides, the featural strength *F*_*ij*_ which was regulated by duration and orientation had a specific input function:


$$F_{i\,j}=\left(1-e^{-d_{i}/b}\right)\,O_{j},$$

where *d* was duration; *b* was a free parameter reflecting growth rate common to all stimuli; and *O* was free parameter that differed for orientations. And the configural strength *C*_*jk*_ was regulated by orientation and stimuli type. According to their results, Loftus et al. (2004) further assumed identical configural strength for upright unfamiliar face and non-face (*C_*UF*_* = *C_*UN*_* = *C*_*U*_), as well as for unfamiliar inverted face and non-face (*C_*IF*_* = *C_*IN*_* = *C*_*I*_), indicating that the configural strength was only affected by orientation in this case. As a result, if we used stimuli type as a state factor, the output functions should become functionally dependent as follows:


$$\left\{ \begin{array}{c} p_{i\,j\,F}=\left[F_{i\,j}+\left(1-F_{i\,j}\right)\,C_{j}\right]\,Y_{F}\\ p_{i\,j\,N}=\left[F_{i\,j}+\left(1-F_{i\,j}\right)\,C_{j}\right]\,Y_{N} \end{array}\right.$$

A straight line with *slope* = *Y_*F*_/Y_*N*_* and *intercept* = *0* would then represent the state trace plot. It was obvious that this multiple-latent-variable model with functionally dependent output mappings could produce a single monotonic curve, but the reverse proposition that the single monotonic state trace plot resulted from a multiple-latent-variable model was not always tenable because, as we had discussed, a single monotonic curve on state trace plot could tell nothing about the number of latent variables. Therefore, it was insufficient to conclude this multiple-latent-variable model if we observed a single monotonic curve, as Loftus et al. (2004) did. Although Loftus et al. (2004) explained their decision to use this model, citing the fact that many researchers held the opinion that face processing relied on two different types of information, this explanation lacked statistical rigor. This model would be supported if we conducted another STA with orientation being the state factor. In this case, the output functions should be:


$$\left\{ \begin{array}{c} p_{i\,U\,k}=\left[F_{i\,U}+\left(1-F_{i\,U}\right)\,C_{U}\right]\,Y_{k}\\ =\left[\left(1-e^{-d_{i}/b}\right)\,O_{j}\,\left(1-C_{U}\right)+C_{U}\right]\,Y_{k}\\ p_{i\,I\,k}=\left[F_{i\,I}+\left(1-F_{i\,I}\right)\,C_{I}\right]\,Y_{k}\\ =\left[\left(1-e^{-d_{i}/b}\right)\,O_{j}\,\left(1-C_{I}\right)+C_{I}\right]\,Y_{k} \end{array}\right.$$

As a result, the relationship between *p*_*iUk*_ and *p*_*iIk*_ was:


$$p_{i\,U\,k}=\frac{O_{U}}{O_{I}}\cdot \frac{1-C_{U}}{1-C_{I}}\,p_{i\,I\,k}+Y_{k}\,\left[C_{U}-\frac{O_{U}}{O_{I}}\cdot \frac{C_{I}\,\left(1-C_{U}\right)}{1-C_{I}}\right]$$

It should be noted that the slope was constant but the intercept was different for *Y*_*k*_, producing parallel lines on the state trace plot. In other words, running the other STA with a different state factor would turn the state trace plot into non-monotonic for this model. Therefore, it was reasonable to assume that this multiple-latent-variable model would be supported if we noticed a non-monotonic state trace plot in the other STA.

Our results demonstrated that the dots on each state trace plot could be well fitted by a single monotonic curve when we used the stimuli type as the state factor, as in earlier studies (Loftus et al., 2004; Prince and Heathcote, 2009). These findings could be explained by either a single-latent-variable structure or a multiple-latent-variable structure with functional dependence. Based on the aforementioned logic, another STA, in which orientation was the state factor, was conducted and produced non-monotonic curves on a state trace plot. These results supported the multiple-latent-variable model and ruled out the possibility of functionally dependent input mappings.

If we adopted Loftus et al. (2004)’s model to explain our results, it was reasonable to assume that *C_*UA*_* = *C_*UC*_* = *C_*UN*_* = *C*_*U*_ and *C_*IA*_* = *C_*IC*_* = *C_*IN*_* = *C*_*I*_ (subscripts *A*, Asian face; *C*, Caucasian face; *N*, Non-face) because of the single monotonic curve for each state trace plot with stimuli type as state factor. Besides, due to the monotonic fitted curves between own- and other-race faces and non-monotonic plots between faces and non-faces when orientation a state factor, it could be inferred that *Y_*A*_=Y_*C*_*≠*Y*_*N*_. In other words, the output functions for own- and other-race faces were exactly the same while for faces and non-faces were different. This was in line with the results of ANOVA where inversion reduced the recognition performance of both own- and other-race faces more significantly than car front, with no difference in performance reduction between the two types of faces. These results were similar to Loftus et al. (2004)’s research where the interaction between orientation and stimuli type (face vs. non-face) was significant but the STA revealed a monotonic curve with stimuli type being a state factor. They argued that FIE did not exist because of the single monotonic state trace plot. However, using the orientation state factor, we found a non-monotonic state trace plot, indicating that different stimuli types (face vs. non-face) differentially influenced recognition performance for upright and inverted images, which could be regarded as the indicator of FIE. Therefore, based on Loftus et al. (2004)’s model, our findings supported the existence of FIE, which was caused by stimuli categories (different Y for faces and non-faces) rather than varying degrees of configural and featural strength utilization. Besides, our results also suggested that there was no difference in the inversion effect between own- and other-race faces (Buckhout and Regan, 1988; Rhodes et al., 2006; Matheson et al., 2012).

Although the model proposed by Loftus et al. (2004) provided a good fit for our results, it should be noted that this model was not the only one available. In a broader sense, our findings for own- and other-race faces could be explained by the fact that, regardless of state factors, the output mappings between them are functionally dependent rather than identical. The ability to recognize different faces in this instance could be attributed to a single virtual latent variable, indicating that the multiple latent variables did not differentially influence the recognition performance (Dunn et al., 2014). Therefore, we could infer that our participants used the same recognition mechanism for unfamiliar own- and other-race faces.

We suggested that there was a single recognition mechanism for both types of faces because, despite the presence of at least two latent variables, such as configural and featural strength (Loftus et al., 2004), they did not differentially affect face recognition and could be collapsed to a single virtual latent variable, such as holistic strength. From this point of view, it implied that the holistic processing—which is crucial for face recognition—was a function of configural and featural information. In fact, according to some researchers, featural information was also crucial for processing upright faces (Cabeza and Kato, 2000) and holistic processing could also exist when processing inverted faces (Richler et al., 2011), suggesting that holistic processing involved more than just the use of configural information (Hayward et al., 2013). However, it should be noted that other studies involving participants from different cultures found varying degrees of inversion between faces of one’s race and those of other races (Rhodes et al., 1989; Sangrigoli and de Schonen, 2004; Hancock and Rhodes, 2008; Megreya et al., 2011). Although the latent variables could not be directly tested by these ANOVA results, they did serve as a reminder that participant race might be a crucial consideration in future research. Given that only Chinese participants were involved in our study and numerous studies had suggested that Asian participants who processed faces more holistically (Blais et al., 2008; McKone et al., 2010) used comparable holistic processing between own- and other-race faces (Michel et al., 2006; Wiese et al., 2009; Mondloch et al., 2010; Crookes et al., 2013; Wong et al., 2021), our results might be exclusive for East Asian participants. This issue required further investigation in future STA studies.

However, there were some limitations to this study. First, as mentioned above, we did not include participants of a different race (e.g., westerners) to see if the same results could also occur in people of a different culture. Our results might be exclusive to Asian participants. Besides, even though all of our participants claimed that they had no direct contact with westerners, given that they were all young college students, it was highly likely that they gained certain experiences with Caucasian faces through the internet. In this information age, it is hard to control this aspect. Enrolling participants who rarely use the internet, such as elderly people living in remote areas, maybe an applicable option. Furthermore, only male faces were used as stimuli in our experiment. Considering that our study involved more female participants who were considered to be better at recognizing their gender faces (Herlitz and Lovén, 2013), the results might be different if female faces were used as stimuli. Finally, we only used unfamiliar face images as stimuli. As a result, we were unable to determine whether the output function of familiar and unfamiliar faces differed. All of these limitations should be taken into account in future STA research.

## Conclusion

Our research using quantitative STA with different state factors supported that at least two latent variables influenced recognition performance in the inversion paradigm. Even though face and non-face stimuli had different effects on the recognition output function, Chinese participants recognized own- and other-race faces in the same way. The findings supported the idea that native Chinese observers used the same recognition mechanism for unfamiliar faces of their race and faces of other races.

## Data availability statement

The original contributions presented in this study are included in the article/supplementary material, further inquiries can be directed to the corresponding author.

## Ethics statement

The studies involving human participants were reviewed and approved by College Research Ethics Committee of Anshun University. The patients/participants provided their written informed consent to participate in this study. Written informed consent was obtained from the individual(s) for the publication of any potentially identifiable images or data included in this article.

## Author contributions

WL designed this study, wrote the draft, and interpreted data. WL and YJ collected and analyzed data. YJ revised the manuscript. Both authors contributed to the article and approved the submitted version.

## Footnotes

1
http://github.com/michaelkalish/STA

2
https://www.autohome.com.cn/

3
https://auto.163.com/
